# Comparative analysis of curcuminoid content, antioxidant capacity, and target-specific molecular docking of turmeric extracts sourced from Thailand

**DOI:** 10.1016/j.fochms.2025.100291

**Published:** 2025-08-25

**Authors:** Shisanupong Anukanon, Komgrit Saeng-ngoen, Yawanart Ngamnon, Ngamnetr Rapan, Weerasak Seelarat, Pannraphat Takolpuckdee, Nisa Pakvilai, Yaiprae Chatree

**Affiliations:** a School of Medicine, Mae Fah Luang University, Chiang Rai 57100, Thailand; b Cancer and Immunology Research Unit (CIRU), School of Medicine, Mae Fah Luang University, Chiang Rai 57100, Thailand; c Faculty of Agricultural Technology, Valaya Alongkorn Rajabhat University under the Royal Patronage, Pathum Thani 13180, Thailand; d Science Center, Valaya Alongkorn Rajabhat University under the Royal Patronage, Pathum Thani 13180, Thailand; e Faculty of Science and Technology, Valaya Alongkorn Rajabhat University under the Royal Patronage, Pathum Thani 13180, Thailand

**Keywords:** Curcumin III, Inflammation, Molecular dynamics

## Abstract

Curcuminoids are the active compounds richest in turmeric rhizomes (*Curcuma longa* L.), comprising curcumin I, demethoxycurcumin (curcumin II), and bisdemethoxycurcumin (curcumin III). This study hypothesized that particular curcumin derivatives could mitigate oxidative stress and inflammation response by targeting specific inflammatory mediators. Therefore, this study aimed to quantify the concentrations of these curcuminoid forms in local turmeric extracts from Thailand. Subsequently, the study analyzed their *in vitro* antioxidant properties, alongside molecular docking and dynamics simulations targeting key oxidative stress- and inflammation-related proteins. Samples were collected from three representative cultivated areas in Thailand: the eastern, southern, and northern regions. The ethanolic extracts from all samples exhibited relatively high total curcuminoid content (eastern: 15.1 %, southern: 25.9 %, and northern: 31.6 % *w*/w in extract), as determined by high-performance liquid chromatography. Curcumin I emerged as the predominant variant, followed closely by curcumin II and III. The ethanolic extracts from the three cultural areas demonstrated significant antioxidant activity, as assessed by ORAC, FRAP, and DPPH assays. Among the three curcuminoids, curcumin III exhibited the strongest predicted binding affinities in molecular docking studies toward antioxidant and anti-inflammatory targets, including 5-LOX, NRF2, IKK1, NF-κB, and NOX4. Molecular dynamics simulations corroborated these findings, revealing that curcumin III formed the most stable complexes, particularly with IKK1, as indicated by low RMSD values (2–3 Å), and high hydrogen bond occupancy. Thus, curcumin III exhibits potential *in silico* inhibition of inflammatory mediators, supporting its promise as a natural compound for antioxidant and anti-inflammatory nutraceutical development.

## Highlights

• Curcumin I was the main variant in turmeric extracts, followed by II and III.
• Curcumin derivatives showed antioxidant capacity relevant to dietary phytochemicals.
• Curcumin III displayed binding affinity to the inflammatory mediator IKK1.

## Graphical abstract

**Figure:**
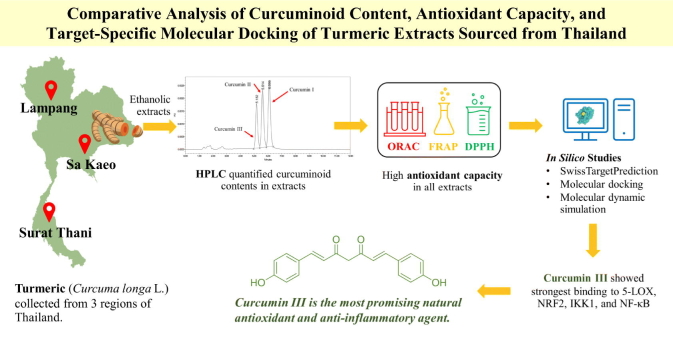

## 1 Introduction

Curcuminoids are bioactive phytochemicals with potent antioxidant properties, widely explored for functional food development (Munekata et al., 2021). These yellow pigments, predominantly concentrated in the rhizome of *Curcuma longa* L. (turmeric) from the Zingiberaceae family, comprise three major components: curcumin I, demethoxycurcumin (curcumin II), and bisdemethoxycurcumin (curcumin III) (Menon & Sudheer, 2007; Priyadarsini, 2014). Their antioxidant activities are valuable for mitigating oxidative damage (Anas et al., 2024).

Oxidative stress, driven by excessive reactive oxygen species (ROS), contributes to chronic diseases such as cardiovascular disorders, neurodegenerative conditions, and cancer through inflammatory signaling and macromolecular damage (Checa & Aran, 2020; Kaur et al., 2020). This process activates pro-inflammatory transcription factors such as NF-κB and impairs antioxidant defenses mediated by the Keap1–NRF2 pathway, which regulates enzymes including superoxide dismutase (SOD) and catalase (Baird & Yamamoto, 2020; Peng et al., 2021). NF-κB signaling, *via* canonical (IKK-mediated IκB degradation) and non-canonical (NIK-mediated p100 processing activated by specific TNF receptor family members) pathways, regulates inflammatory gene expression in response to cytokines, growth factors, and pathogens (Mao & Zhao, 2025; Yu et al., 2020). Similarly, cyclooxygenase-2 (COX-2) catalyzes proinflammatory prostaglandin synthesis at inflammation sites, while NRF2 upregulates antioxidant enzymes such as NAD(*P*)H:quinone oxidoreductase 1 (NQO1) and heme oxygenase 1 (HO-1) (Faki & Er, 2021; Jaiswal, 2004).

Curcumin derivatives modulate these pathways by inhibiting COX-2, 5-lipoxygenase (5-LOX), and NADPH oxidase 4 (NOX4), as well as upstream regulators such as IκB kinase (IKK) (Derochette et al., 2013; Goel et al., 2001). However, curcumin's low solubility, poor bioavailability, and rapid metabolism limit its efficacy, necessitating investigation of natural derivatives with potentially improved biological profiles (Anand et al., 2007; Lagoa et al., 2025).

Despite extensive research on curcumin's bioactivity, comparative analyses of curcumin I, II, and III interactions with a comprehensive set of oxidative stress and inflammation-related targets remain limited. This study aims to: (1) quantify curcumin I, II, and III concentrations in local turmeric extracts using high-performance liquid chromatography (HPLC); and (2) evaluate their binding affinities to NF-κB, IKK1, COX-2, 5-LOX, NOX4, NRF2, SOD, and catalase *via* molecular docking. This integrative approach, which predicts ligand-target interactions with high precision, provides novel insights into the molecular mechanisms of curcumin derivatives, supporting their potential development as dietary compounds for reducing chronic disease risk at the population level.

## 2 Materials and methods

### 2.1 Chemicals and reagents

All solvents used for extraction and experiments in this study were of analytical grade and obtained Thermo Fisher Scientific, Milford, USA. High-performance liquid chromatography (HPLC)-grade solvents were utilized for HPLC mobile phases in active compound identification. A mixture of curcumin standards (curcumin, demethoxycurcumin, and bisdemethoxycurcumin, 98 % purity) was purchased from Thermo Fisher Scientific, Milford, USA.

### 2.2 *Plant material and extraction of Curcuma longa* L*.*

Rhizomes of *Curcuma longa* L. were obtained from three different cultivated areas in Thailand: Sa Kaeo province, Surat Thani province, and Lampang province. Samples were collected from these representative local cultivated areas, including the Eastern region (Sa Kaeo province), the Southern region (Surat Thani province), and the Northern region (Lampang province). The collected samples from Sa Kaeo, Surat Thani, and Lampang were planted at (13.4219^°^ N, 102.3372^°^ E, altitude 213.36 nautical miles), (8.9245^°^ N, 98.91^°^ E, altitudes 36.45 nautical miles), (18.99^°^ N, 99.79^°^ E, altitude 732.51 nautical miles), respectively. The samples were collected during November–December 2024, and their taxonomy was identified by the Department of Agricultural Management Technology, Faculty of Agricultural Technology, Valaya Alongkorn Rajabhat University under the Royal Patronage, Pathum Thani, Thailand. The bulked sample from each site was thoroughly mixed and randomly subdivided into three biological replicates. In total, 9 replicates were cleaned and subsequently oven-dried at 60 °C for 8 h until a constant weight was achieved. Dried samples (100 g each) were then used for extraction in individual batches. For each assay, analyses were performed in triplicate to ensure technical replication.

### 2.3 Curcuminoid extraction

One hundred grams of dried samples were extracted using ethanolic extraction. The solvent-to-solid ratio was 1:8 (*w*/*v*), and the extraction was performed for 24 h on a stirrer. The ethanol solvents were removed under vacuum using a rotary evaporator (Buchi, Flawil, Switzerland). The ethanolic extracts were kept in the dark at 4 °C until use.

### 2.4 Determination of active compounds using high performance liquid chromatography

Quantification of curcuminoids was carried out using high-performance liquid chromatography with UV/Visible detection (Waters e2695 separations module, Milford, USA). A reverse-phase C18 column (4.6 × 150 mm, i.d. 5 μm particle size; GL Sciences, Tokyo, Japan) was employed. Quantitative concentrations of curcuminoids were determined by injecting a volume of 20 μL of the sample at a flow rate of 0.8 mL/min and detecting the absorbance at 425 nm. Samples were prepared through ethanol extraction followed by filtration using a 0.45 μm membrane. The mobile phase consisted of a mixture of 2 % acetic acid in water and acetonitrile at a 50:50 (*v*/v) ratio under isocratic condition. The concentrations of each curcuminoid were reported by calculation of its standard curve.

### 2.5 Determination of total polyphenol content

The total polyphenol content (TPC) of the ethanolic extract was determined using the Folin-Ciocalteu spectrophotometric method (Lu et al., 2007). Total phenol content was expressed as milligrams of gallic acid equivalent (GAE) per gram of dry matter extract (mg GAE/g dw).

### 2.6 Determination of antioxidant properties

#### 2.6.1 Oxygen radical absorbance capacity (ORAC)

Hydrophilic chain-breaking antioxidant activity was assessed using the oxygen radical absorbance capacity (ORAC) assay (Ou et al., 2001). Briefly, a fluorescence probe was employed to detect the free radical trapping capacity of the ethanolic extracts. A loss of fluorescence intensity at an excitation wavelength of 485 nm and an emission wavelength of 528 nm was measured using spectrophotometry. The antioxidant activity of Trolox was used to create a standard calibration curve. ORAC capacity was reported as micromoles of Trolox equivalents (TE).

#### 2.6.2 Ferric reducing antioxidant power (FRAP)

The ferric reducing ability of plasma was assessed using the ferric reducing antioxidant power (FRAP) assay (Benzie & Strain, 1996). This determination is based on the reduction of ferric (Fe^3+^) to ferrous (Fe^2+^) ions at low pH, resulting in the blue color of the ferrous- tripyridyl triazine complex. Absorbance was measured at 593 nm. The total FRAP value was calculated based on its calibration curve and reported as micromoles of Trolox equivalents (TE).

#### 2.6.3 DPPH radical scavenging activity

The radical scavenging activity of the sample was evaluated using 1,1-diphenyl-2- picrylhydrazyl (DPPH) radical scavenging assay (Katsube et al., 2004). The radical scavenging activity of the ethanolic extracts was determined by adding DPPH solution to the extracts in each well of a 96-well plate. Color development was measured spectrophotometrically at a wavelength of 550 nm, and the scavenging activity was reported in millimoles of Trolox equivalents (TE). The percentage of inhibition against the concentration of the ethanolic extract was also expressed as IC_50_ values.

### 2.7 Computational target prediction

The chemical structures of curcumin I, II, and III were uploaded to the SwissTargetPrediction site (Swiss Institute of Bioinformatics, 2025; http://www.swisstargetprediction.ch/, accessed March 22, 2025) to forecast probable targets based on chemical similarities. The species was designated as *Homo sapiens*. The probability values of the predicted targets were documented for each compound. Targets exhibiting high probability scores associated with oxidative stress and inflammation, such as NF-κB, IKK1, NOX4, COX-2, 5-LOX, NRF2, SOD, and catalase, were selected for subsequent molecular docking studies.

#### 2.7.1 Molecular docking analysis

The crystal structures of target proteins were obtained from the Protein Data Bank (PDB) and processed by removing all water molecules, solvents, and co-crystallized ligands using the ViewerLite tool (Accelrys, San Diego, USA). The chosen targets included 5-LOX (PDB ID: 3V99), COX-2 (4 M11), NOX4 (4UT2), catalase (1DGG), SOD (1HL5), NF-κB (1SVC), NRF2 (8IVG), and IKK1 (5EBZ) (Gilbert et al., 2011; Müller et al., 1995; Otake et al., 2023; Polley et al., 2016; Putnam et al., 2000; Santos et al., 2016; Strange et al., 2003; Xu et al., 2014). Energy-minimized structures of curcumin I, II, and III were separately docked into the active sites of each target using AutoDock 4.2.6 with default parameters. A molecular grid with a spacing of 0.375 Å was centered on the active site, with a grid box size of 60 × 60 × 60 points to encompass the binding pocket. Protein structures were prepared by removing water molecules and adding polar hydrogens, while ligands were energy-minimized and set as flexible during docking. For each ligand, 10 independent docking runs were performed using the Lamarckian Genetic Algorithm, and binding affinities (ΔG) were estimated using AutoDock's scoring function (Morris et al., 2009). Binding free energies (BE, kcal/mol) and estimated inhibitory constants were computed for all docking conformations. The optimal binding poses were identified based on minimal energy values and visually assessed using UCSF Chimera software version 1.15 (Pettersen et al., 2004). The software was obtained from the UCSF Resource for Biocomputing, Visualization, and Informatics (2004) (https://www.cgl.ucsf.edu/chimera/; accessed August 7, 2025).

#### 2.7.2 Molecular dynamics (MD) simulation

Molecular dynamics (MD) simulations were conducted using GROMACS 2021.4 with the CHARMM36 force field to further examine the stability of the ligand–protein complexes (Abraham et al., 2015). Only the optimal docking poses (minimum binding free energy) of Curcumin I, II, and III bound to their respective targets were selected for simulation. Each complex was placed within a defined TIP3P water box featuring periodic boundary conditions (Vega & de Miguel, 2007). Energy minimization was performed using the steepest descent method, followed by system equilibration under constant volume (NVT) and constant pressure (NPT) ensembles for 50 ps each. The production run was conducted for 100 ns at 310 K, utilizing the V-rescale thermostat for temperature regulation and the Parrinello-Rahman barostat for pressure coupling (Hoover, 1985; Nosé, 1984; Parrinello & Rahman, 1981). Trajectory analyses were performed to compute the root mean square deviation (RMSD) of the ligand–protein complexes to evaluate overall stability, while minimum distance (mindist) analyses were performed to assess enduring interactions between the ligands and critical amino acid residues throughout the simulation.

## 3 Results and analysis

### 3.1 Quantitative analysis of the major curcuminoid constituents

**Fig. 1:**
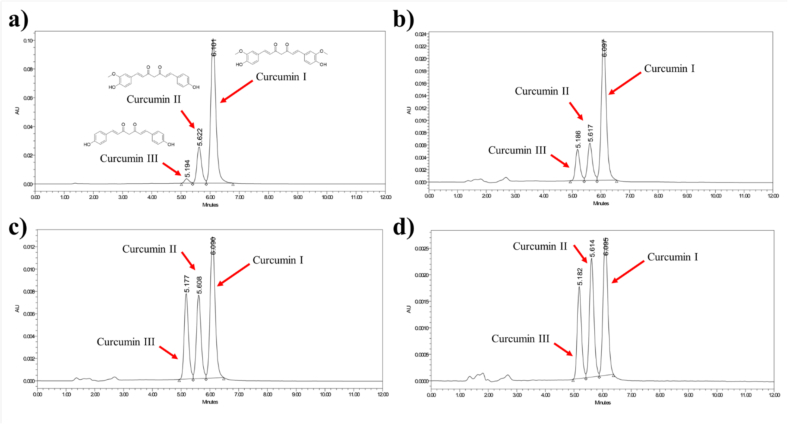
Representative HPLC chromatograms of curcuminoids. (a) Chromatogram of the standard curcuminoid mixture showing retention times for curcumin III (∼5.19 min), curcumin II (∼5.62 min), and curcumin I (∼6.10 min). (b–d) Chromatograms of ethanolic turmeric extracts from (b) Lampang, (c) Surat Thani, and (d) Sa Kaeo provinces. All three curcuminoids were identified in each extract, with variations in peak intensity and distribution indicating differences in curcuminoid composition among regions.

The percentage yields of ethanolic extracts from *Curcuma longa* L. sourced from Sa Kaeo, Lampang, and Surat Thani provinces were 8.26 %, 9.26 %, and 12.46 %, respectively. The highest extractable yield was derived from the Surat Thani sample (638.09 g fresh weight, producing 79.48 g of dried extract), followed by Lampang (41.40 g from 447.15 g), and Sa Kaeo (18.65 g from 225.83 g). The yield variances may indicate differences in regional cultivation conditions, rhizome maturity, or post-harvest processing. HPLC analysis was conducted to identify and quantify the major curcuminoid constituents in the ethanolic extracts namely, curcumin I, curcumin II, and curcumin III. This was accomplished by comparing the retention times of the constituents to those of authenticated standards using a UV detector set at 425 nm (Fig. 1). Identification was based on comparison with known retention times of standard compounds, which were approximately 5.19 min for curcumin III, 5.62 min for curcumin II, and 6.10 min for curcumin I (Fig. 1a). All three curcuminoids were detected in the extracts from all locations, although the relative peak intensities varied, reflecting differences in curcuminoid composition among the samples. Notably, the Lampang extract displayed the most prominent peak for curcumin I, while the extracts from Sa Kaeo and Surat Thani exhibited a more balanced distribution across the three curcuminoids (Fig. 1b-d). These variations correspond with the quantitative findings and are further explored in the sections discussing antioxidant activity and computational analysis. The contents of individual curcuminoids were expressed as grams of compound per 100 g of extract, serving as a key indicator of the pharmacological potential of each turmeric sample.

The percentage compositions of curcuminoids in the ethanolic extract of three different cultural areas of local turmeric (*Curcuma longa* L.) were determined by HPLC analysis in accordance with their standard calibration curve. Compounds were reported as curcumin I, II, and III and summarized in Table 1. The results showed that curcumin I was the major compound present in the ethanolic extracts among the three forms of curcuminoids. Sa Kaeo contained relatively low percentages of curcumin I, II, and III at 11.67 %, 2.90 %, and 0.52 % *w*/w in extract, respectively. The total curcuminoid content in the ethanolic extract of the Sa Kaeo group was found to be 15.10 % w/w. Surat Thani showed moderate percentage of curcumin I, II, and III at 18.58 %, 5.50 %, and 1.85 % w/w in extract, respectively, with a total curcuminoid content of 25.93 ± 0.03 % w/w. In contrast, Lampang exhibited the greatest amount of curcuminoids, with percentages of curcumin I, II, and III at 25.64 %, 4.74 %, and 1.27 % w/w in extract, respectively. The total curcuminoid content of the Lampang group was 31.65 ± 0.28 % w/w. This finding indicates that the dominant curcuminoids in the ethanolic extracts from the three cultivated areas are curcumin I, followed by curcumin II and III. The highest average percentage of total curcuminoids was found in the sample from the representative cultivated area in northern Thailand. These results are consistent with previous reports that curcumin is the major curcuminoid component in the extract of *Curcuma longa* L. (Jayaprakasha et al., 2002; Pothitirat & Gritsanapan, 2005). The relatively high proportion of curcuminoids in our samples suggests potential for enhanced antioxidant activity.

**Table 1:** Composition of curcuminoids from three different cultural areas of *Curcuma longa* L. (% *w*/w in extract) grams per 100 g of the extract.

Curcuminoids	Sample			
	Sa Kaeo	Surat Thani	Lampang	*p*-value
Curcumin I	11.67 ± 0.00	18.58 ± 0.02	25.64 ± 0.07	< 0.05
Curcumin II (Demethoxycurcumin)	2.90 ± 0.00	5.50 ± 0.01	4.74 ± 0.19	< 0.05
Curcumin III (Bisdemethoxycurcumin)	0.52 ± 0.00	1.85 ± 0.00	1.27 ± 0.02	< 0.05
Total curcumin	15.10 ± 0.00	25.93 ± 0.03	31.65 ± 0.28	< 0.05

The numbers represent the average percentage (*w*/w in extract) of three replications.

Statistically significant between-group comparisons were performed by ANOVA following Turkey's multiple comparisons test at *p* < 0.05.

### 3.2 Total phenolic contents and antioxidant activities

Total phenolic contents and antioxidant activity of three ethanolic extracts from representative local cultivars of *Curcuma Longa* L. in Thailand are summarized in Table 2. The ethanolic extracts from samples collected in Sa Kaeo, Surat Thani, and Lampang exhibited relatively high total phenolic content (TPC) at 219.53 ± 12.93, 172.43 ± 9.35, and 201.90 ± 5.28 mg eq. GA/g, respectively (Table 2). The polyphenol compounds in these extracts conferred significant antioxidant activity, as chain-breaking antioxidant activity determined by the ORAC assay, was measured at 7204.22 ± 254.89, 7375.87 ± 227.91, and 7307.96 ± 296.27 μmoles TE for samples from Sa Kaeo, Surat Thani, and Lampang, respectively, without significant differences observed between the groups. The antioxidant power (FRAP) of the ethanolic extract from Sa Kaeo (1387.33 ± 16.92 μmoles TE) was significantly higher than that of Surat Thani (1241.47 ± 10.93 μmoles TE) but comparable to that of Lampang (1352.95 ± 45.23 μmoles TE). Thus, the relatively high reducing potential of the three tested samples indicates strong antioxidant properties. There were no statistically significant differences in antioxidant activity among the sample collection areas as indicated by the ORAC assay.

**Table 2:** Total phenolic contents and antioxidant activity of the ethanolic extracts.

	Sample		
	Sa Kaeo	Surat Thani	Lampang
Total phenolic contents (mg eq. GA/g)	219.53 ± 12.93^a^	172.43 ± 9.35^b^	201.90 ± 5.28^ab^
Antioxidant activity			
ORAC (μmoles TE)	7204.22 ± 254.89	7375.87 ± 227.91	7307.96 ± 296.27
FRAP (μmoles TE)	1387.33 ± 16.92^a^	1241.47 ± 10.93^b^	1352.95 ± 45.23^ab^
DPPH (mmoles TE)	811.20 ± 9.96^a^	755.38 ± 35.71^a^	1013.07 ± 20.69^b^

Values are derived from three replicated experiments (Mean ± S.D.).

Different superscripts ^**(**a, b, c)^ indicate statistical significance (*p* < 0.05).

The extracts from the three different groups also demonstrated relatively high radical scavenging capacity, as determined by the DPPH assay. The radical scavenging capacity of the ethanolic extract from Lampang showed the significantly higher than that of the other groups, measuring 1013.07 ± 20.69 mmoles TE, compared to Sa Kaeo (811.20 ± 9.96 mmoles TE) and Surat Thani (755.38 ± 35.71 mmoles TE) (Table 2). The percentage of DPPH inhibition for the ethanolic extracts from the three different cultural areas of local turmeric (*Curcuma longa* L.) is shown in Suppl. Fig. 1. Sa Kaeo exhibited an inhibition concentration of 50 % DPPH (IC_50_ values) at 21.22 μg/mL, while Surat Thani showed an IC_50_ of 14.38 μg/mL. Lampang presented the lowest IC_50_ value at 9.35 μg/mL. All ethanolic extracts effectively scavenged DPPH^•^ free radicals in a concentration-dependent manner. Lampang, a sample from the northern region of Thailand, has the most potent radical scavenging activity, characterized by hydrogen atom transfer (HAT) as defined by the DPPH assay. Curcuminoids in the ethanolic extracts from the three different cultural areas exhibited scavenging activity. The particular curcuminoids, main ingredients might be responsible for the active compounds involved in radical chain-breaking activity and relief from oxidative damage. However, other phenolic compounds in the extracts might influence significant differences in antioxidant activities between samples. Overall, the ethanolic extract of *Curcuma longa* L. from the three different areas demonstrated high effectiveness in antioxidant properties defined by hydrogen atom transfer, electron donation, and reducing power mechanism.

### 3.3 Computational target prediction

To identify potential molecular targets linked to the antioxidant and anti-inflammatory properties of Curcumin I, II, and III, SwissTargetPrediction was employed. The heatmap (Suppl. Fig. 2) illustrates the anticipated probabilities, focusing on key targets associated with oxidative stress and inflammation, such as NRF2, IKK1, 5-LOX, NOX4, iNOS, COX-1, COX-2, TNF-α, and STAT3. The heatmap revealed that curcumin III exhibited the highest predicted probabilities for key antioxidant-related targets, showing significant binding probabilities for NRF2 (0.2173), 5-LOX (0.2173), COX-1 (0.2173), and IKK1 (0.1852), and was uniquely projected to interact with NOX4 (0.1133), a major ROS-producing enzyme. Curcumin II exhibited moderate probabilities for NRF2 (0.1140), 5-LOX (0.1475), and COX-1 (0.1140), and indicated interactions with iNOS (0.0972) and TNF-α (0.0972), implying a potential role in inflammatory modulation. Curcumin I exhibited comparable but lower probabilities (0.1016) across NRF2, 5-LOX, COX-1, and IKK1, with no anticipated interaction with NOX4, iNOS, or TNF-α.

### 3.4 Molecular docking analysis

The molecular docking results demonstrated that curcumin III consistently exhibited the highest binding affinities across various targets (Table 3). It showed the most favorable binding affinities to 5-LOX (−10.25 kcal/mol, *Ki* 30.76 nM), NRF2 (−9.16 kcal/mol, *Ki* 194.37 nM), IKK1 (−9.73 kcal/mol, *Ki* 73.57 nM), and NF-κB (−8.39 kcal/mol, *Ki* 711.17 nM). Curcumin III also displayed substantial binding affinity for NOX4 (−7.04 kcal/mol, *Ki* 6.93 μM), an essential enzyme involved in the generation of reactive oxygen species. Curcumin II exhibited moderate binding energies, particularly toward 5-LOX (−9.26 kcal/mol, *Ki* 162.32 nM) and IKK1 (−8.00 kcal/mol, *Ki* 1.36 μM), while curcumin I demonstrated marginally lower binding affinities across all targets, with the weakest interaction noted against NOX4 (−5.23 kcal/mol, *Ki* 146.39 μM). The docking results closely aligned with the SwissTargetPrediction probabilities, indicating that curcumin III exhibited the greatest potential for robust interactions with principal antioxidant and inflammatory targets, followed by curcumin II and curcumin I.

**Table 3:** Molecular docking analysis of curcumin I, II, and III toward antioxidant and inflammatory targets.

Target (PDB ID)	Binding energies, BE (kcal/mol) [estimated inhibitory constants, *K*_*i*_]		
	Curcumin I	Curcumin II	Curcumin III
**5-LOX** (3V99)	−9.07 [224.13 nM]	−9.26 [162.32 nM]	−10.25 [30.76 μM]
**COX-2** (4M11)	−9.36 [137.63 nM]	−9.16 [194.27 nM]	−9.53 [103.15 nM]
**NOX4** (4UT2)	−6.44 [19.14 μM]	−5.23 [146.39 μM]	−7.04 [6.93 μM]
**Catalase** (1DGG)	−6.63 [13.71 μM]	−6.19 [29.16 μM]	−6.38 [21.00 μM]
**SOD** (1HL5)	−6.81 [10.23 μM]	−7.18 [5.45 μM]	−7.19 [5.38 μM]
**NF-κB** (1SVC)	−7.70 [2.25 μM]	−6.97 [7.76 μM]	−8.39 [711.17 nM]
**NRF2** (8IVG)	−8.89 [306.01 nM]	−7.93 [1.54 μM]	−9.16 [194.37 nM]
**IKK1** (5EBZ)	−8.59 [505.78 nM]	−8.00 [1.36 μM]	−9.73 [73.57 nM]

### 3.5 Molecular dynamics (MD) simulation

5-LOX, NF-κB, NRF2, and IKK1 were selected as representative targets in this study due to their central roles in inflammatory and oxidative stress pathways. Following molecular docking, MD simulations were conducted on these targets to further assess the binding stability and interaction patterns of curcumin I, II, and III.

**Fig. 2:**
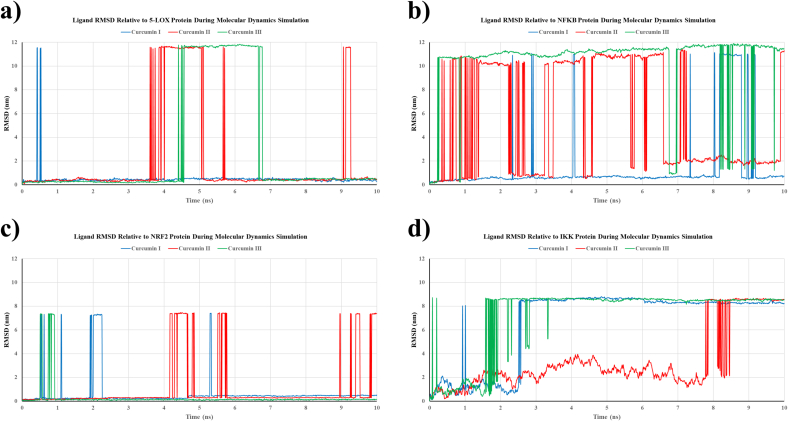
The root-mean-square deviation (RMSD) profiles of curcumin–protein complexes during 10-ns MD simulations. The RMSD profiles for curcumin I (blue), curcumin II (orange), and curcumin III (green) complexed with 5-LOX (a), NF-κB (b), NRF2 (c), and IKK (d) were monitored over the simulation period. These plots illustrate the ligand backbone deviations relative to the initial docked positions, indicating the binding stability of each curcuminoid to its respective protein target over time. (For interpretation of the references to color in this figure legend, the reader is referred to the web version of this article.)

The ligand RMSD trajectories of curcumin I, II, and III complexed with 5-LOX, NF-κB, NRF2, and IKK1 were analyzed during a 10 ns MD simulation. Within the 5-LOX complex, curcumin III and curcumin I displayed remarkably stable RMSD profiles (RMSD between 0.2 and 0.3 nm or 2–3 Å), while curcumin II exhibited significant instability with frequent substantial deviations (>10 nm) prior to re-equilibration (Fig. 2a). In the NF-κB complex, curcumin I exhibited the most stable binding (RMSD 0.2–0.3 nm), whereas curcumin II and III demonstrated significant variations, indicating weaker or less consistent binding (Fig. 2b). Within the NRF2 complex, all three curcumins exhibited commendable stability with slight variations, although curcumin II occasionally manifested spikes (Fig. 2c). In the IKK1 complex, curcumin III and I achieved steady RMSD values of approximately 0.8–0.9 nm following early fluctuations, while curcumin II exhibited comparatively lower stability with wider oscillations during the simulation (Fig. 2d).

**Fig. 3:**
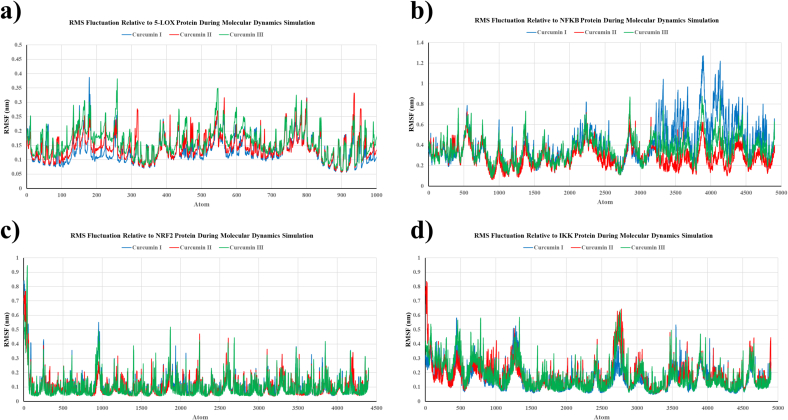
Root-mean-square fluctuation (RMSF) profiles of amino acid residues in curcumin–protein complexes. The RMSF profiles demonstrate the flexibility of amino acid residues in 5-LOX (a), NF-κB (b), NRF2 (c), and IKK proteins (d) during 10-ns molecular dynamics simulations after binding with curcumin I, II, and III. Lower RMSF values at binding sites suggest stabilization of protein structures by the curcuminoids, while higher fluctuations indicate regions of structural flexibility.

The root-mean-square fluctuation (RMSF) profiles were analyzed to assess the flexibility and local motion of amino acid residues in each protein following curcumin binding during the MD simulation. In the 5-LOX complex, all curcuminoids exhibited relatively minimal fluctuations, predominantly ranging from 0.1 to 0.3 nm, with curcumin III showing somewhat greater fluctuations in flexible loop regions (Fig. 3a). Significant changes (∼0.5–1.2 nm) were observed in key residue areas of the NF-κB complex, particularly in the curcumin I complex, indicating dynamic structural modifications (Fig. 3b). The NRF2 complex displayed minimal changes across all curcumin variants, generally remaining below 0.3 nm, signifying a well-stabilized protein structure (Fig. 3c). Moderate changes (∼0.2–0.6 nm) were noted in the IKK1 complex, with slight variations among the three forms of curcumin. Overall, the RMSF results indicated that curcumin III marginally enhanced local flexibility in active regions while preserving global protein stability (Fig. 3d).

**Fig. 4:**
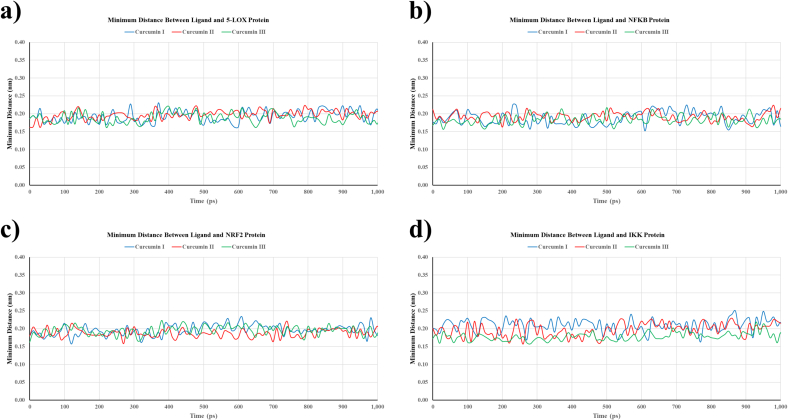
Minimum distance analysis between curcuminoids and protein targets during molecular dynamics simulations. The minimum distance plots show the closest distance (in nm) between each curcuminoid (curcumin I, II, III) and their respective protein binding sites (5-LOX, NF-κB, NRF2, IKK) over 10 ns. Stable minimum distance values indicate persistent ligand–protein interactions, reflecting the stability of the binding during the simulation period.

The minimum distance (mindist) between each curcumin derivative and their corresponding antioxidant-related protein targets remained consistently between 0.17 and 0.22 nm during the 10 ns simulation period. For 5-LOX, curcumin III exhibited the narrowest and most constant minimum distance (∼0.17 nm), indicating a strong binding affinity (Fig. 4a). For NF-κB, all curcumins displayed comparable distances (∼0.18–0.21 nm), although curcumin III demonstrated marginally closer contact (Fig. 4b). The minimum distance for all three curcumins in relation to NRF2 varied significantly but remained approximately 0.18–0.20 nm, signifying persistent interactions (Fig. 4c). For IKK1, curcumin III exhibited the nearest average distance (∼0.17 nm), while curcumin I and II presented slightly greater distances (Fig. 4d).

**Fig. 5:**
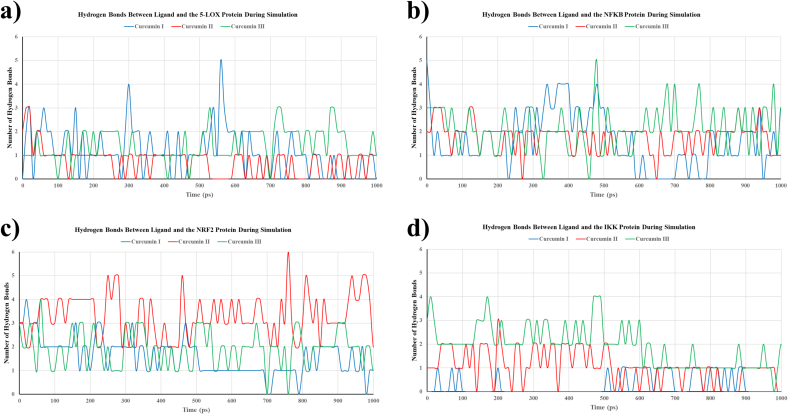
illustrates the number of hydrogen bonds formed between curcuminoids and protein targets during molecular dynamics simulations. The analysis of hydrogen bond formation between curcumin I, II, and III and their corresponding protein targets (5-LOX, NF-κB, NRF2, IKK) throughout the 10-ns simulation reveals fluctuations in hydrogen bond numbers that reflect the dynamic nature of ligand–protein interactions. Higher and more consistent hydrogen bonding indicates stronger and more stable binding.

The hydrogen bond analysis between the three curcumin derivatives and the selected targets during MD simulations revealed distinct interaction patterns. In the 5-LOX complex, curcumin I and curcumin III exhibited fluctuating hydrogen bond numbers, ranging from 0 to 5 throughout the simulation, while curcumin II maintained a relatively stable bond number, primarily between 1 and 2 (Fig. 5a). In the NF-κB system, curcumin III maintained more consistent hydrogen bond formation compared to curcumin I and II, although all compounds formed 1–5 hydrogen bonds intermittently. Curcumin II displayed slightly lower average bond counts (Fig. 5b). For the NRF2 protein, curcumin II exhibited the highest number of hydrogen bonds, occasionally forming up to six bonds, while curcumin I and III maintained lower, steadier bond counts (2–3 bonds) (**Figure _5c**). In the IKK1 complex, curcumin III demonstrated superior hydrogen bond formation relative to curcumin I and II, with curcumin I displaying the lowest number of hydrogen bonds with IKK1, including several periods where no bonds were detected (Fig. 5d).

## 4 Discussion

Ethanolic extracts from local turmeric *(Curcuma longa* L*.)* grown in three different regions demonstrated a strong ability to scavenge free radicals, as evidenced by their high antioxidant capacity in ORAC, FRAP, and DPPH tests. The antioxidant capacity is associated with the total phenolic content, highlighting the role of curcuminoids in mitigating oxidative stress. Among the curcuminoid contents, curcumin I is the predominant curcuminoid, followed closely by demethoxycurcumin (curcumin II) and bisdemethoxycurcumin (curcumin III). These findings are consistent with previous reports by Tanvir et al. (2017), which demonstrated the potent antioxidant activity of ethanolic extracts from local turmeric (*Curcuma longa* L*.*) in Bangladesh, particularly regarding high antioxidant properties and polyphenol levels (Tanvir et al., 2017). The ethanolic extract exhibited DPPH radical-scavenging activity with a 50 % inhibitory concentration (IC_50_) of 1.08 μg/mL and also showed a relatively high reducing power determined by the FRAP value (Tanvir et al., 2017). Although the curcuminoid concentration in the extracts from Sa Kaeo is half that of Lampang, curcuminoids still demonstrate potent antioxidant activity through the ferric reducing antioxidant power mechanism. However, the significant difference of curcuminoid concentrations between Sa Kaeo and Lampang shows the difference in radical scavenging capacity, as determined by the DPPH assay. This discrepancy suggests that while Sa Kaeo's extracts may have lower curcuminoid concentrations, they still possess unique properties that could contribute to their overall antioxidant efficacy. The recent study has reported that curcuminoids extracted from turmeric showed strong antioxidant activity by reducing power at low concentration (Stasiłowicz-Krzemień et al., 2024). However, the matrix effect, which involves a complex mixture of various components such as terpenoids, synergistic phytochemicals, and other polyphenols, may affect the antioxidant effect (Chen et al., 2022).

In comparison to another study, the turmeric extract from samples collected in the northern region of Thailand had a total curcuminoid content of approximately 81 % *w*/w in the extract (curcumin I 54 %, curcumin II 16 %, and curcumin III 11 %). The extract from the southern region contained a total curcuminoid content of approximately 90 % w/w (curcumin I 60 %, curcumin II 17 %, and curcumin III 13 %) (Pothitirat & Gritsanapan, 2005). However, the curcuminoid concentrations were influenced by the different extraction methods (*e.g.*, Soxhlet apparatus) and analysis techniques (*e.g.*, TLC method). This variation underscores the importance of selecting appropriate techniques for extracting and analyzing curcuminoids to ensure accurate and reliable results. Therefore, future studies should focus on optimizing these methods to enhance the yield and purity of the compounds. Additionally, the concentrations of curcuminoids in turmeric are influenced by environmental conditions and soil quality in which it is grown (Silaru et al., 2024). Curcuminoid concentrations in turmeric are highly variable, influenced by a complicated combination of environmental factors (*e.g.*, climate, rainfall, sunlight) and soil quality (*e.g.*, pH, nutrient profiles) specific to different cultivation regions (Sandeep et al., 2015). To control these ecological factors may be advantageous to the standardization of curcuminoid yield in the turmeric plant.

Dietary antioxidants, including curcuminoids, play a critical role in reducing the risk of chronic diseases at the population level through incorporation into habitual dietary patterns (Dehzad et al., 2023; Peng et al., 2021). Oral administration of 1 g of curcuminoids and 10 mg of piperine daily for eight weeks significantly increased superoxide dismutase (SOD) levels and decreased malondialdehyde (MDA) concentrations in patients with metabolic syndrome compared to a placebo group (Panahi et al., 2015). A randomized, double-blind, placebo-controlled trial had shown that curcumin supplementation at 1500 mg per day for 10 weeks in patients with type 2 diabetes mellitus significantly reduced high-sensitivity C-reactive protein (CRP) levels compared to the control group (Adibian et al., 2019). Supplementation with 500 mg turmeric capsules, containing 22 mg of curcumin as the active ingredient, in type 2 diabetic patients with end-stage renal disease (ESRD) due to nephropathy for two months significantly lowered serum levels of TGF-β and IL-8, as well as urinary protein excretion (Khajehdehi et al., 2011). These findings underscore the clinical relevance of curcuminoids in managing oxidative stress and inflammation-related conditions.

The antioxidant capacity of food-derived bioactive compounds contributes to reducing disease risk when incorporated into habitual dietary patterns at the population level. The physiological effects of these compounds vary according to their digestion, absorption, metabolism, and the health status of the individual. *In vitro* assays such as DPPH (2,2-diphenyl-1-picrylhydrazyl), FRAP (ferric reducing antioxidant power), and ORAC (oxygen radical absorbance capacity) quantify the radical-scavenging capacity of food matrices, enabling standardized comparisons across studies. However, oxidative stress in biological systems also involves non-radical reactive species and dysregulation of endogenous redox systems. Many bioactives, including polyphenols and curcuminoids, confer health benefits through mechanisms beyond direct radical scavenging, such as modulation of inflammatory signaling pathways, regulation of enzyme activity, and alterations in gene expression. These bioactives are consistent with public health recommendations from the World Health Organization (WHO) and national dietary guidelines, which advocate for the consumption of plant-based foods rich in phytochemicals to promote long-term health and reduce the risk of noncommunicable diseases (Hossain et al., 2025; Leitzmann, 2016; World Health Organization, 2021).

Antioxidant assays provide standardized methods for evaluating the radical-scavenging capacity of food-derived compounds. The Dietary Antioxidant Index (DAI), which expresses the ratio of dietary antioxidant intake to recommended values, has been inversely associated with obesity-related conditions such as type 2 diabetes and dyslipidemia (Pourmontaseri et al., 2024). Higher DAI scores have also been correlated with reduced oxidative stress, lower levels of pro-inflammatory mediators, and decreased risk of cancer and cardiovascular mortality (Wang & Yi, 2022). Recent evidence further indicates that higher DAI is inversely associated with both the prevalence of nonalcoholic fatty liver disease (NAFLD) and cancer mortality risk in patients with NAFLD (Ding et al., 2025). Although the WHO has established an acceptable daily intake (ADI) for curcumin of 3 mg/kg body weight, the DAI specific to curcumin remains unreported (Andrekowisk Fioravanti et al., 2024). Clinical studies suggest that curcumin supplementation may beneficially modulate risk factors for atherosclerotic cardiovascular disease, including systolic and diastolic blood pressure, LDL cholesterol, TNF-α, malondialdehyde (MDA) levels, and HDL cholesterol in patients with type 2 diabetes mellitus (El-Rakabawy et al., 2025). Furthermore, dietary curcumin may reduce oxidative stress markers in both diabetic and nondiabetic patients with proteinuric chronic kidney disease (Jiménez-Osorio et al., 2016). While these findings provide mechanistic plausibility, population-level benefits are more appropriately assessed through long-term dietary studies rather than single compound supplementation trials.

Computational research has clarified the molecular basis of these bioactivities. The integration of computational target prediction, molecular docking, and MD simulations indicates that curcumin III is the most promising variant among the three. Although curcumin I and II initially exhibited higher probability scores in SwissTargetPrediction for various classical inflammation-related targets, including 5-LOX, IKK1, and NF-κB, the experimental robustness of these interactions was further substantiated through molecular docking and MD simulations. Curcumin III demonstrated the most robust and sustained binding to key oxidative and inflammatory targets, including 5-LOX, NRF2, IKK1, and NF-κB. This discovery suggests that although predictive techniques are useful for screening options, molecular docking and dynamic simulations are crucial for evaluating the true binding affinity and interactions of compounds with their targets.

The molecular docking findings indicated that curcumin III exhibited the most advantageous binding energies across multiple targets, demonstrating sub-micromolar inhibitory capability against 5-LOX, NRF2, and IKK1. 5-LOX is a crucial enzyme that facilitates leukotriene synthesis and inflammatory reactions, while IKK1 is vital for stimulating NF-κB signaling, leading to the generation of several inflammatory cytokines (Gilbert et al., 2012; Huxford et al., 1998). The strong binding affinity to NRF2 further supports the function of curcumin III in enhancing antioxidant defense, potentially through the regulation of cellular redox homeostasis (Ashrafizadeh et al., 2020).

MD simulations confirmed the enhanced stability of curcumin III compared to curcumin I and II. The stable RMSD profiles and continuous minimal distances indicated that curcumin III maintained consistent engagement with its targets throughout the simulation period. The ability to maintain persistent binding interactions is essential for the biological efficacy of small compounds, particularly when interacting with flexible protein domains like the transcription factors NF-κB and NRF2 (Gunasekaran & Nussinov, 2007). Curcumin III demonstrated superior binding and stability relative to curcumin I, despite having fewer methoxy groups. This can be attributed to the enhanced accommodation of the smaller, less voluminous curcumin III molecule within the binding sites of these targets. A more compact and adaptable structure may enable curcumin III to conform more easily to various protein environments and sustain effective interactions. The hydrogen bond analysis confirmed the enhanced interaction stability of curcumin III. The persistent establishment of hydrogen bonds with essential residues in proteins such as IKK1 and NF-κB supports the notion that curcumin III may achieve enhanced inhibitory effects by stabilizing critical regions of these proteins. Notably, although curcumin II occasionally formed a greater number of hydrogen bonds, these interactions did not consistently lead to improved overall complex stability, highlighting the significance of both hydrogen bond quality and overall molecular fit.

Curcumin I, II, and III, while closely related, exhibit significant chemical variations that affect their binding characteristics, stability, and biological activity. All three compounds share a common β-diketone linker connecting two aromatic rings; however, the differing substitution patterns on the aromatic rings significantly influence their molecular behavior (Ravindranath & Satyanarayana, 1980; Ruby et al., 1995). Curcumin I contains two methoxy (-OCH₃) groups and two hydroxyl (-OH) groups located at the 3- and 4-positions of each phenyl ring. Curcumin II (demethoxycurcumin) is characterized by the absence of one methoxy group on an aromatic ring, while curcumin III (bisdemethoxycurcumin) lacks both methoxy groups, retaining only the hydroxyl groups. The gradual removal of methoxy groups results in nuanced yet significant alterations in electronic distribution, molecular dimensions, and polarity. The presence of methoxy groups in curcumin I enhances the electron-donating capacity of the aromatic rings, promoting molecular conjugation and slightly increasing its hydrophobicity. This enhanced delocalization may facilitate curcumin I's interaction with hydrophobic pockets in specific proteins, but it also leads to a larger, more rigid conformation. This bulkiness may restrict its ability to conform to smaller or more adaptable protein binding sites. In contrast, curcumin III, devoid of methoxy groups, is less voluminous and exhibits greater flexibility. The absence of methoxy groups reduces steric hindrance around the aromatic rings, allowing curcumin III to more readily adapt to various binding environments. Although curcumin III exhibits slightly reduced hydrophobicity compared to curcumin I, its smaller size and enhanced flexibility appear to facilitate superior accommodation within diverse protein pockets, as evidenced by its elevated binding affinity and stability in MD simulations. These findings indicate that the therapeutic benefits of turmeric extracts are not solely dependent on curcumin I but are also significantly influenced by curcumin III, which may serve as a precursor for future advancements in antioxidant and anti-inflammatory nutraceuticals.

However, our findings are significantly limited by the inherent physicochemical properties of Curcumin III, specifically its poor water solubility, restricted bioavailability, and rapid metabolism and elimination. Curcuminoids, being lipophilic compounds, demonstrate poor solubility that directly impedes their systemic availability (Wolnicka-Glubisz & Wisniewska-Becker, 2023). This condition leads to rapid metabolism and elimination of Curcumin III, drastically limiting its systemic exposure and sustained therapeutic efficacy (Fança-Berthon et al., 2021). Although these characteristics pose a significant obstacle to therapeutic application, they also underscore the necessity of innovative delivery systems. To enhance systemic exposure and pharmacodynamic efficiency, future research should investigate advanced formulation strategies, including liposomal carriers, nanoparticle encapsulation, and bioenhancer co-administration (Dehzad et al., 2023). Consequently, while Curcumin III may exhibit promising molecular mechanisms, overcoming its inherent bioavailability challenges will be crucial. To translate these *in vitro* discoveries into clinically relevant outcomes, further investigation will be necessary, especially through rigorous clinical trials that focus on physiological absorption, distribution, metabolism, and excretion.

Despite molecular docking and molecular dynamics (MD) simulations have offered valuable insights into the binding affinity and stability of curcumin III with critical oxidative and inflammatory targets, these computational methods are inherently limited. These computational methods are conducted under idealized and simplified conditions that do not accurately represent the complexity of biological systems. *In silico*, factors such as protein conformational flexibility, metabolic transformation, cellular transport, and systemic interactions are not completely captured (Gunasekaran & Nussinov, 2007; Pronk et al., 2013). Consequently, our results indicate that curcumin III has robust and consistent interactions with targets such as 5-LOX, NRF2, and IKK1, but these interactions should be regarded as predictive rather than conclusive. In order to verify the therapeutic potential and physiological relevance of these computationally derived interactions, it is imperative to conduct experimental validation using *in vitro* and *in vivo* models.

Furthermore, curcumin III's clinical significance remains unexplored. The majority of curcuminoids have been the subject of most extant research, with curcumin I being the primary focus. However, there has been relatively little investigation into the specific effects of curcumin III, particularly in human subjects. While informative, the current evidence is primarily derived from molecular docking, cell-based assays, or preclinical animal models, which do not completely capture the intricacies of human physiology. Thus, it is imperative to conduct well-designed, large-scale clinical trials that evaluate the pharmacokinetics, therapeutic efficacy, safety, and tolerability of curcumin III in order to verify its practical application. Additionally, the standardized composition of extracts and the development of more effective delivery systems are essential prerequisites for the successful translation of curcumin III into dietary-based approaches or nutraceutical applications.

In conclusion, the empirical antioxidant capacity assessed by ORAC, FRAP, and DPPH assays reinforces the assertion that extracts of *Curcuma longa* L*.,* particularly those rich in curcumin III and related compounds, may provide substantial protection against oxidative damage. This highlights the necessity of integrating chemical profiling, biological testing, and computational predictions to comprehensively evaluate the therapeutic potential of natural compounds, which may serve as precursors for future breakthroughs in antioxidant and anti-inflammatory pharmaceuticals and nutraceuticals.

## CRediT authorship contribution statement

**Shisanupong Anukanon:** Writing – original draft, Methodology, Formal analysis, Conceptualization. **Komgrit Saeng-ngoen:** Investigation, Funding acquisition, Conceptualization. **Yawanart Ngamnon:** Investigation. **Ngamnetr Rapan:** Investigation. **Weerasak Seelarat:** Investigation. **Pannraphat Takolpuckdee:** Funding acquisition. **Nisa Pakvilai:** Funding acquisition. **Yaiprae Chatree:** Writing – review & editing, Writing – original draft, Methodology, Investigation, Funding acquisition, Data curation, Conceptualization.

## Declaration of generative AI and AI-assisted technologies in the writing process

During the preparation of this work, the authors used ChatGPT and QuillBot to correct grammar during manuscript revision. After using these tools, the authors reviewed and edited the content as needed and took full responsibility for the publication.

## Declaration of competing interest

The authors declare that they have no known competing financial interests or personal relationships that could have appeared to influence the work reported in this paper.

## Data Availability

Data will be made available on request.

## Appendix A Supplementary Data

Supplementary Fig. 1 Percentage of DPPH inhibition for the three different ethanolic turmeric extracts Supplementary Fig. 1

Supplementary Fig. 2 Heatmap of target probabilities for curcumin I, II, and III Supplementary Fig. 2
