# Supplementary material for: Comparative analysis of curcuminoid content, antioxidant capacity, and target-specific molecular docking of turmeric extracts sourced from Thailand
Source: Food Chem (Oxf). 2025 Aug 25;11:100291. doi: 10.1016/j.fochms.2025.100291 (PMC12418845; doi:10.1016/j.fochms.2025.100291)
Supplement: Supplementary Fig. 1 — Percentage of DPPH inhibition for the three different ethanolic turmeric extracts [file mmc1.docx]

**(****Suppl. Fig.1)**


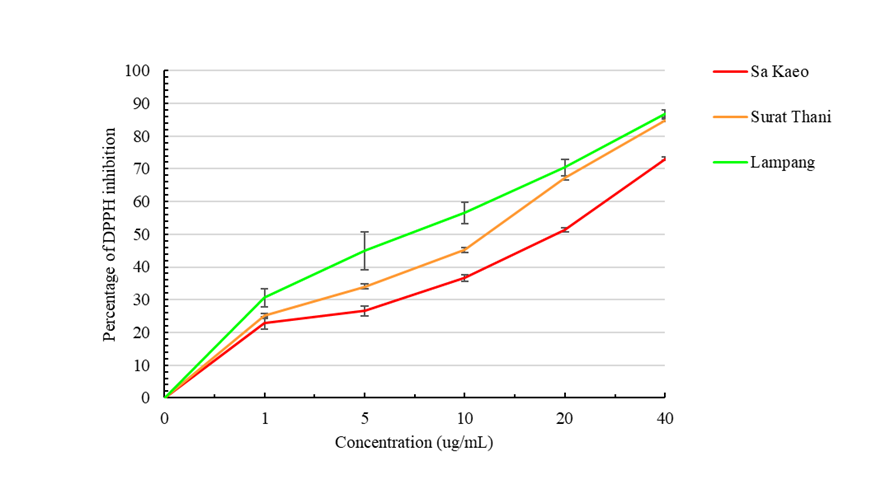


**Suppl. Fig.1** Percentage of DPPH inhibition for the three different ethanolic turmeric extracts
