# Supplementary material for: Comparative analysis of curcuminoid content, antioxidant capacity, and target-specific molecular docking of turmeric extracts sourced from Thailand
Source: Food Chem (Oxf). 2025 Aug 25;11:100291. doi: 10.1016/j.fochms.2025.100291 (PMC12418845; doi:10.1016/j.fochms.2025.100291)
Supplement: Supplementary Fig. 2 — Heatmap of target probabilities for curcumin I, II, and III [file mmc2.docx]

**Suppl. Fig.2**


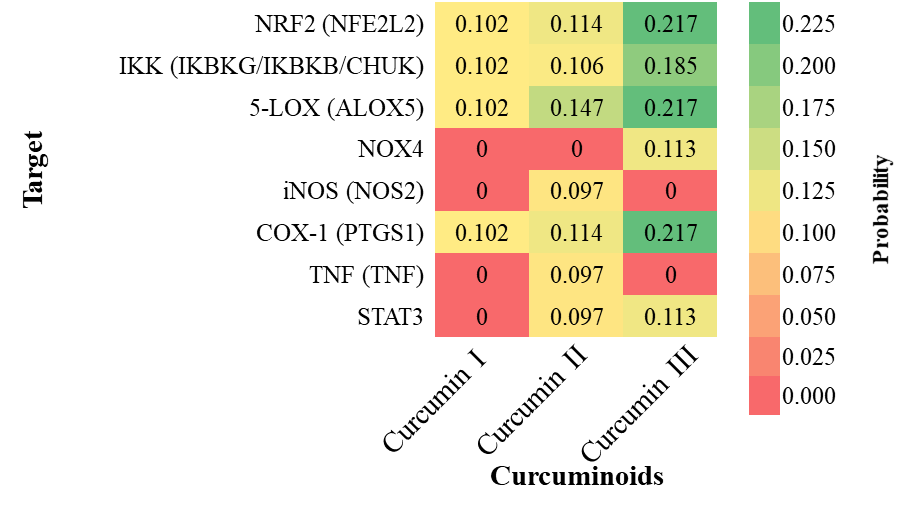


**Suppl. Fig.2** Heatmap of target probabilities for curcumin I, II, and III

(green = high probability, yellow = intermediate probability, red = low probability)
